# Association between serum 25-hydroxyvitamin D₃ and retinol in Chinese children (2019–2023): an isoform-specific cross-sectional analysis

**DOI:** 10.3389/fnut.2026.1985336

**Published:** 2026-10-15

**Authors:** Yue Bi, Jiaxing Yi, Qiao Wang, Hehe Chen, Jishan Zheng, Shanshan Wang, Wenyuan Liu

**Affiliations:** 1 Laboratory of Medicine, Ningbo Zhenhai People's Hospital Group, Ningbo, Zhejiang, China; 2 School of Medicine, Ningbo University, Ningbo, Zhejiang, China; 3 Laboratory of Medicine, Qiu'ga Central Health Clinic, Ningbo, Zhejiang, China; 4 Ningbo Key Laboratory for the Prevention and Treatment of Embryo Original Diseases, The Affiliated Women and Children’s Hospital of Ningbo University, Ningbo, Zhejiang, China; 5 Department of Infectious Diseases, The Affiliated Women and Children's Hospital of Ningbo University, Ningbo, Zhejiang, China; 6 Ningbo Key Laboratory of Genomic Medicine and Birth Defects Prevention, The Affiliated Women and Children's Hospital of Ningbo University, Ningbo, Zhejiang, China; 7 School of Public Health, Zhejiang University, Hangzhou, Zhejiang, China

**Keywords:** 25-hydroxyvitamin D3, children, China, dose–response, non-linear, public health, retinol, subgroup analysis

## Abstract

**Background:**

China’s national pediatric supplementation program exclusively uses vitamin D₃ (cholecalciferol). Yet, the association between circulating 25-hydroxyvitamin D₃ [25(OH)D₃] and retinol in Chinese children has not been fully characterized with isoform-specific assays in a large, temporally expanded cohort. This study aimed to characterize the non-linear dose–response relationship between serum 25(OH)D₃ and retinol and to examine how this association varies across demographic and seasonal strata.

**Methods:**

We retrospectively analyzed 6,774 children aged 0–10 years who underwent routine health surveillance across 22 community centers in Ningbo, China (2019–2023). Serum 25(OH)D₃ was resolved separately from D₂ by liquid chromatography–tandem mass spectrometry. Restricted cubic splines (RCS), multivariable linear regression, and bootstrap-validated (*n* = 1,000) non-linear modeling were used to characterize the D₃–retinol association. Subgroup analyses by sex, age, season, and D₃ stratum were visualized using forest plots.

**Results:**

Mean serum 25(OH)D₃ was 30.9 ± 11.1 ng/mL and mean retinol was 272.5 ± 80.6 ng/mL. A significant non-linear, saturable relationship was evident (*p*-overall < 0.001; *p*-non-linearity <0.001). In the lower repletion range, each 1 ng/mL increment in D₃ corresponded to a 3.16 ng/mL rise in retinol (95% CI: 2.66–3.65; *p* < 0.001); as D₃ increased, the slope progressively attenuated to 1.80 (95% CI: 1.46–2.15; *p* < 0.001) but remained positive. Bootstrap resampling confirmed a stable transition zone centered at 28.7 ng/mL (95% CI: 26.4–31.2). The association was strongest in winter (*β* = 2.70; 95% CI: 2.23–3.17), with significant seasonal heterogeneity (*p* for interaction = 0.002); the point estimate was largest in children aged 6–10 years (*β* = 4.10; 95% CI: 2.10–6.11), although the age-by-D₃ interaction was not significant (*p* = 0.358), and this subgroup comprised only 93 children (1.4%).

**Conclusion:**

Serum 25(OH)D₃ exhibits a non-linear, isoform-specific association with retinol, with the steepest relationship observed in the insufficient-to-sufficient transition range. Maintaining D₃ within optimal repletion—particularly during winter—may support retinol status for bone development and mucosal immunity.

## Introduction

Retinol and vitamin D are fat-soluble micronutrients whose concerted actions govern pediatric skeletal mineralization, mucosal barrier integrity, and immune tolerance. Despite decades of fortification, concurrent insufficiency remains prevalent in Chinese children, particularly during rapid growth phases when metabolic demands peak and dietary transitions occur (1, 2). Crucially, multi-regional surveys across China have documented that co-deficiency of both vitamins is far from rare, suggesting that single-nutrient assessments frequently underestimate the true public health burden (3).

Vitamin D₃ (cholecalciferol) constitutes the dominant circulating isoform in humans, typically accounting for >90% of total 25-hydroxyvitamin D [25(OH)D] in East Asian populations where ergocalciferol (D₂) intake is negligible. Compared with D₂, D₃ exhibits higher binding affinity for vitamin D-binding protein (DBP), preferential 25-hydroxylation by hepatic CYP2R1, and a longer circulating half-life, conferring greater molar potency and metabolic stability (4, 5). Because China’s national pediatric supplementation program delivers D₃ exclusively—and because D₂ contribution to serum 25(OH)D is practically nil in this population—total 25(OH)D assays effectively average a potent, abundant isoform with a negligible one. Consequently, D₃-specific thresholds may offer superior clinical resolution for guiding supplementation and predicting micronutrient status.

Vitamin A (retinol) and D₃ are frequently co-administered in pediatric practice because both are essential for bone health and immune function (6). Epidemiological studies have reported parallel seasonal fluctuations and age-related gradients for both vitamins, yet the precise shape of their dose–response relationship in children remains poorly defined (7, 8). Whether the association is uniform across the physiological range or exhibits differential strength at varying D₃ concentrations is clinically relevant for determining supplementation targets.

Our group previously reported a non-linear association between total 25(OH)D and retinol in 4,752 Ningbo children aged 0–14 years (2018–2021), with an apparent change in slope near 28.3 ng/mL (9). However, that analysis relied on total 25(OH)D assays that did not chromatographically resolve D₃ from D₂, leaving unresolved whether the observed pattern was driven by the metabolically dominant D₃ isoform or diluted by the weaker D₂ signal. Moreover, the broad age range (0–14 years) and analytical focus on a dichotomous breakpoint limited characterization of the continuous dose–response landscape. To address these gaps, we conducted the present investigation in an expanded cohort of 6,774 children aged 0–10 years (2019–2023), using isoform-specific LC–MS/MS quantification to isolate the D₃ signal and modeling the D₃–retinol relationship as a continuous non-linear association. This design enables us to re-examine, in an isoform-specific framework, whether the previously observed total-D pattern reflects a true D₃-specific phenomenon and to characterize the consistency and shape of this association across sex, age, and season.

## Materials and methods

This study followed the STROBE (Strengthening the Reporting of Observational Studies in Epidemiology) guidelines for the transparent reporting of observational research.

### Study design and population

Between 1 January 2019 and 31 December 2023, we retrospectively identified children aged 0–10 years who had attended routine preventive health surveillance at 22 community health-service centers across Ningbo’s Yinzhou and Zhenhai districts. We explicitly note that the two studies partially overlap: of the 6,774 children in the present dataset, 3,409 (50.3%) were sampled between 2019 and 2021 and therefore fall within the sampling frame of our previous study (9); (2018–2021, *n* = 4,752). Because both datasets were fully anonymized without a common identifier, individual-level linkage between the two datasets was not possible; accordingly, the 3,409 figure represents the maximum potential overlap, and the exact number of children included in both studies cannot be determined. This expanded center network was designed to improve precision for isoform-specific estimation. The age range was deliberately restricted to 0–10 years to capture pre-pubertal children during peak windows of skeletal growth and dietary transition, while excluding older adolescents in whom hormonal changes and divergent lifestyle patterns might confound the D₃–retinol association.

This study was conducted in accordance with the ethical principles of the Declaration of Helsinki. The study protocol was approved by the Ethics Committee of the Affiliated Women and Children’s Hospital of Ningbo University in 2025 (Approval No. NBFE-2025-KY-203) for the retrospective analysis of anonymized specimens and de-identified surveillance data generated during routine care between 2019 and 2023. Given the retrospective nature of the data analysis, the requirement for informed consent was waived by the ethics committee.

Inclusion required attendance at the health-examination session and absence of chronic disorders known to alter vitamin absorption or bone metabolism. To preserve D₃-specific interpretability, we applied two additional exclusion criteria not used in the earlier total-D analysis: (i) use of pharmacological vitamin D₂ or mixed D₂/D₃ supplements within the preceding 4 weeks; and (ii) acute infection or inflammation within 4 weeks (C-reactive protein >10 mg/L or clinician-documented febrile illness), because retinol is an acute-phase reactant. Children with chronic kidney or liver disease, malabsorption syndromes, hyperparathyroidism, or current use of anticonvulsants, glucocorticoids, or rifampin were also excluded.

### Measurement of 25(OH)D₃ and retinol

Fasting venous blood was collected, centrifuged within 2 h, and sera stored at −20 °C for centralized batch analysis. Serum 25(OH)D₃ was quantified separately from 25(OH)D₂ by liquid chromatography–tandem mass spectrometry (AB SCIEX Triple Quad 4500MD) using a Kinetex C18 column (2.6 μm, 100 × 2.1 mm; Phenomenex). The mobile phase comprised 0.1% formic acid in water (A) and methanol (B), delivered at 0.4 mL/min with a linear gradient: 70% B at 0 min, rising to 95% B by 4.0 min, held until 5.0 min, then re-equilibrated. Multiple-reaction monitoring transitions were m/z 401.3 → 257.2 for 25(OH)D₃ and m/z 413.3 → 269.2 for the deuterated internal standard. 25(OH)D₂ was resolved with a retention-time difference >0.4 min, ensuring isoform-specific quantification. Serum retinol was measured concurrently on the same platform. Daily quality control employed certified reference materials (Hefei Hehe Medical Technology); intra-assay coefficients of variation were 4.2% (D₃) and 3.8% (retinol); inter-assay coefficients were 6.1 and 5.5%, respectively (*n* = 15 runs).

This isoform-specific approach contrasts with the total 25(OH)D assay used in our prior 2018–2021 analysis, which could not distinguish D₃ from D₂ and therefore reported a combined signal in which the weaker D₂ isoform may have diluted the metabolic contribution of D₃ (9).

D₃ nutritional status was classified as: severe deficiency (≤10 ng/mL), deficiency (>10–20), insufficiency (>20–30), sufficiency (>30–100), and excess (>100). Retinol status was judged against age-stratified reference intervals (0–6 years: 113–647 ng/mL; 7–12 years: 128–812 ng/mL), with deficiency defined as below the lower limit.

### Statistical analysis

Continuous variables were summarized as mean ± standard deviation; categorical variables as counts and column percentages. Group differences were evaluated via parametric tests (two-sample *t* or ANOVA) where distributional assumptions held; otherwise, non-parametric alternatives were applied.

Dose–response characterization and threshold identification. The D₃–retinol relationship was first visualized with restricted cubic splines, and departure from linearity was tested with a Wald *χ*^2^ statistic. Threshold effects were then estimated with two-piecewise linear regression; the inflection point was identified by grid search over the 10th–90th percentile range and confirmed with a likelihood ratio test comparing the segmented model against a simple linear fit. Bootstrap resampling (1,000 iterations) was applied to derive bias-corrected 95% confidence intervals for the threshold location.

Association analyses and subgroup heterogeneity. Multivariable linear regression was performed with two levels of covariate adjustment: a crude model (unadjusted) and an adjusted model controlling for age (continuous), sex, calendar year of enrollment (to account for secular trends including COVID-19-related behavioral changes), and season of blood draw. Subgroup analyses by sex, age group, and season were conducted by fitting separate adjusted models within each stratum; Wald tests for product terms between continuous D₃ and each stratification variable were used to generate p-for-interaction values. For the threshold-stratified panel of the forest plot, separate linear regression models were fitted within each D₃ stratum (<28.9 and ≥28.9 ng/mL), with D₃ centered at the subgroup mean to improve numerical stability. These stratum-specific estimates are presented alongside the primary piecewise regression results for visual complementarity.

Forest plots were used to visualize subgroup-specific effect sizes and their precision. All analyses used R version 4.3.1 and Free Statistics software v2.1.2. Two-sided *p* < 0.05 denoted significance.

## Results

### Baseline characteristics

Table 1 delineates the baseline characteristics of the 6,774 participants, stratified by serum 25(OH)D₃ status with embedded retinol metrics. The cohort included 14 (0.2%) children with severe deficiency, 350 (5.2%) with deficiency, 672 (9.9%) with insufficiency, and 5,738 (84.7%) with sufficiency. Overall, 51.9% were male, and the median age was 0.7 years (IQR: 0.6–1.5). Mean serum D₃ was 30.9 ± 11.1 ng/mL, and mean retinol was 272.5 ± 80.6 ng/mL.

**Table 1:** Descriptive characteristics of participants by 25(OH)D₃ status categories.

Variables	Total (*n* = 6,774)	Severe deficiency or deficiency (*n* = 364)	Insufficiency (*n* = 672)	Sufficiency (*n* = 5,738)	*p*
Sex, *n* (%)					0.508^b^
Male	3,514 (51.9)	193 (53.0)	335 (49.9)	2,986 (52)	
Female	3,260 (48.1)	171 (47.0)	337 (50.1)	2,752 (48)	
Age, years, Median (IQR)	0.7 (0.6, 1.5)	1.2 (0.6, 3.4)	1.0 (0.6, 3.0)	0.7 (0.6, 1.1)	<0.001^a^
Season, *n* (%)					<0.001^c^
Spring	1950 (28.8)	155 (42.6)	223 (33.2)	1,572 (27.4)	
Summer	2,157 (31.8)	112 (30.8)	232 (34.5)	1813 (31.6)	
Autumn	1,524 (22.5)	40 (11.0)	106 (15.8)	1,378 (24)	
Winter	1,143 (16.9)	57 (15.7)	111 (16.5)	975 (17)	
25(OH)D₃, ng/mL, Mean ± SD	30.9 ± 11.1	11.6 ± 3.0	17.7 ± 1.4	33.6 ± 9.6	<0.001^a^
Retinol, ng/mL Mean ± SD	272.5 ± 80.6	224.4 ± 66.8	241.4 ± 74.3	279.3 ± 80.2	<0.001^a^
Retinol status					<0.001^c^
Retinol deficiency, *n* (%)	44 (0.6)	12 (3.3)	13 (1.9)	19 (0.3)	
Retinol normal or excess, *n* (%)	6,730 (99.4)	352 (96.7)	659 (98.1)	5,719 (99.7)	

IQR, interquartile range; Mean ± SD, mean ± standard deviation. “a” refers to the one-way ANOVA test; “b” refers to the Chi-square test; “c” refers to the Fisher’s exact test.

A marked age gradient was observed across D₃ categories (*p* < 0.001). Children in the combined severe deficiency/deficiency group were older (median 1.2 years, IQR: 0.6–3.4) than those in the sufficiency group (median 0.7 years, IQR: 0.6–1.1). The seasonal distribution of blood sampling also varied significantly (*p* < 0.001): 42.6% of the combined severe/deficiency group were sampled in spring versus 27.4% of the sufficient group, reflecting the seasonal nadir of cutaneous D₃ synthesis after winter UVB deprivation.

The lower panel of Table 1 details retinol status distribution. Overall, 44 children (0.6%) were retinol deficient, while 6,730 (99.4%) maintained normal or supranormal concentrations. Mean retinol levels rose in tandem with D₃ status, from 224.4 ± 66.8 ng/mL in the severe/deficiency group to 279.3 ± 80.2 ng/mL in the sufficiency group (*p* < 0.001). The prevalence of retinol deficiency also declined across improving D₃ strata: 3.3% in the severe/deficiency group, 1.9% in the insufficiency group, and 0.3% in the sufficiency group (*p* < 0.001). Among the 44 retinol deficient children, 63.6% were female, the median age was 0.5 years (IQR: 0.5–0.7), and 45.5% were sampled in summer—patterns consistent with younger age and seasonal dietary transitions observed in this cohort.

### Non-linear dose–response

RCS analysis revealed a significant non-linear, saturable relationship between serum 25(OH)D₃ and retinol (*p*-overall <0.001; *p*-non-linearity <0.001; Figure 1). The dose–response curve exhibited a steep ascending phase at lower D₃ concentrations, followed by a gradual attenuation of slope as D₃ increased, consistent with a non-linear rather than simple linear association.

**Figure 1:**
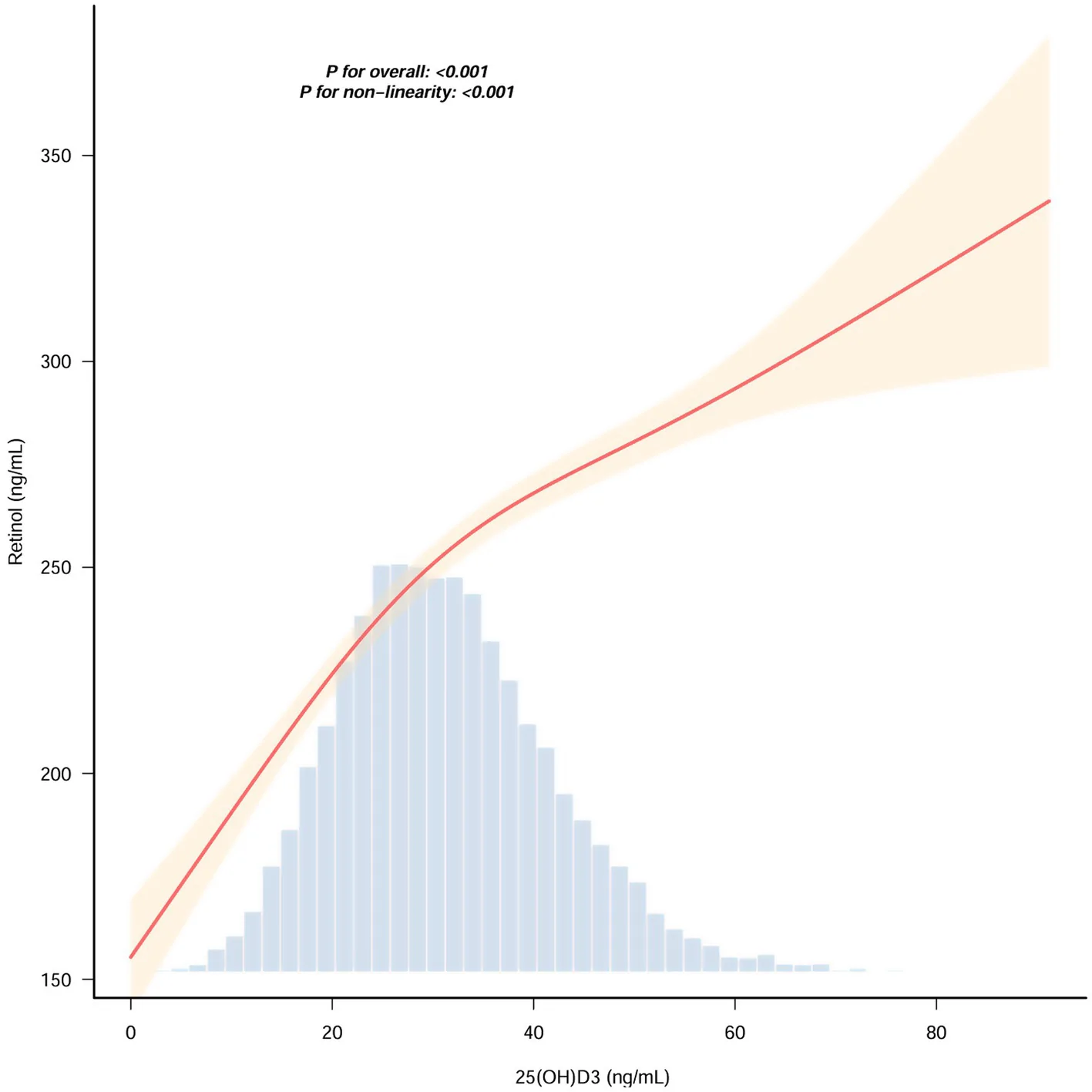
Restricted cubic spline plot of the multivariable-adjusted association between serum 25-hydroxyvitamin D₃ and retinol. The solid line depicts the predicted mean retinol concentration (ng/mL) across the D₃ distribution, with dashed lines representing 95% confidence intervals. The model is adjusted for age, sex, calendar year, and season. The curve demonstrates a steep ascending phase at lower D₃ concentrations with progressive attenuation of slope at higher concentrations, consistent with a non-linear saturable pattern.

Quantitative modeling identified a transition region centered at 28.9 ng/mL (Table 2). where the strength of the D₃–retinol association markedly changed character. Below this transition zone, each 1 ng/mL increment in D₃ corresponded to a 3.158 ng/mL rise in retinol (95% CI: 2.661–3.654; *p* < 0.001); above this region, the slope attenuated to 1.804 (95% CI: 1.457–2.151; *p* < 0.001) but remained statistically positive, indicating that the association persists even at higher D₃ concentrations, albeit with diminished marginal returns. Bootstrap resampling (1,000 iterations) yielded a median transition point of 28.7 ng/mL (95% CI: 26.4–31.2), confirming robustness of the saturation profile.

**Table 2:** Threshold effect analysis of the relationship between 25(OH)D₃ and retinol.

25(OH)D₃	*β* (95% CI)	*p* value
<28.9 ng/mL	3.158 (2.661, 3.654)	<0.001
≥28.9 ng/mL	1.804 (1.457, 2.151)	<0.001
Likelihood ratio test	–	<0.001

*β* (95% CI), unstandardized regression coefficient (95% confidence interval); adjusted for age, sex, calendar year, and season.

### Association between 25(OH)D₃ and retinol across subgroups

Figure 2 presents the multivariable-adjusted association between serum 25(OH)D₃ and retinol as *β* coefficients with 95% confidence intervals across clinically relevant subgroups. In the overall analysis, each 1 ng/mL increment in D₃ was associated with a 2.11 ng/mL rise in retinol after adjustment for age, sex, calendar year, and season (95% CI: 1.94–2.28; *p* < 0.001), representing a 12% increase from the crude estimate (*β* = 1.88; 95% CI: 1.72–2.05).

**Figure 2:**
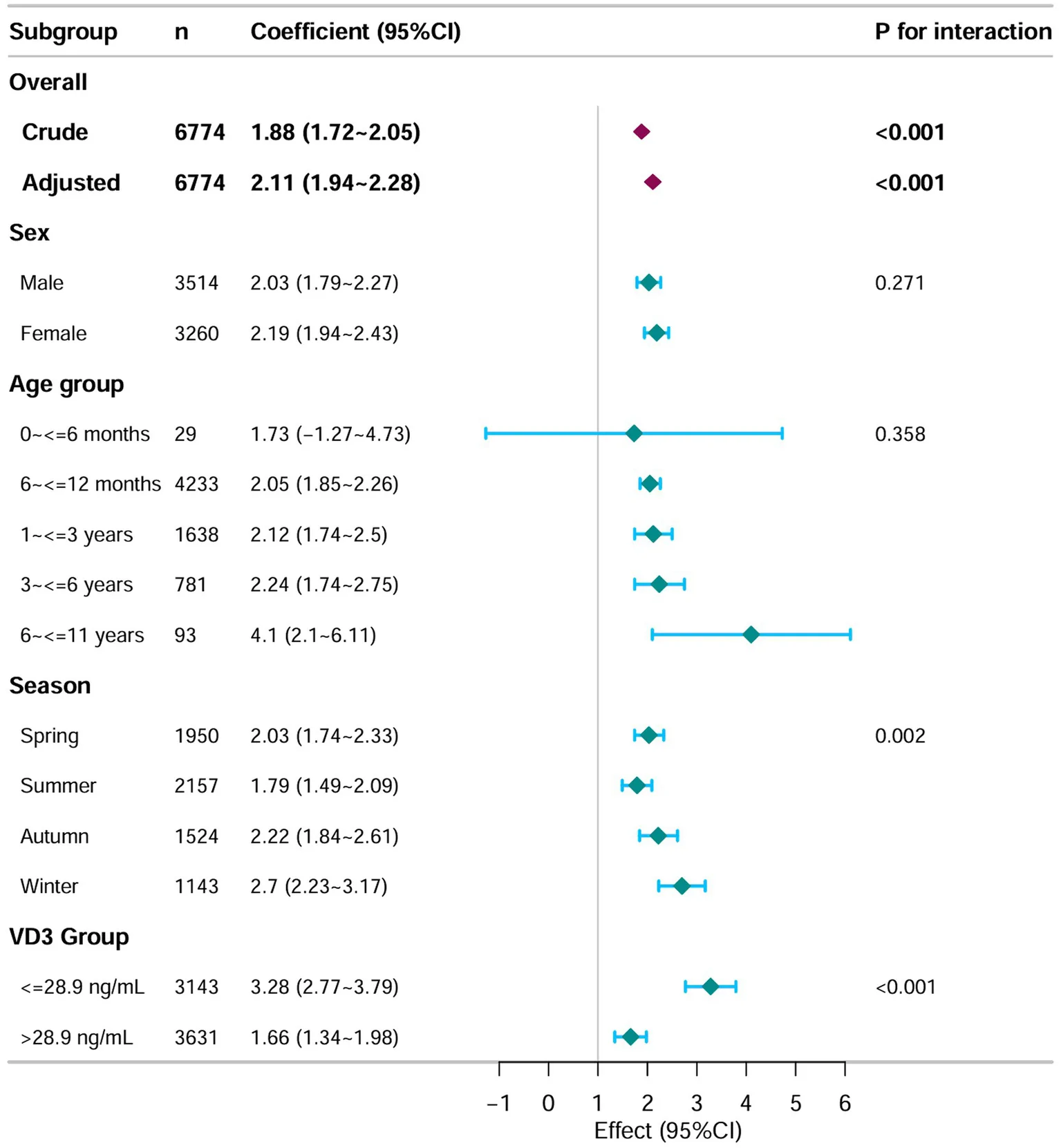
Forest plot of the multivariable-adjusted association between serum 25-hydroxyvitamin D₃ and retinol across subgroups. Effect sizes are presented as *β* coefficients (95% confidence intervals) for the change in retinol (ng/mL) per 1 ng/mL increase in D₃, derived from linear regression models adjusted for age, sex, calendar year, and season (except where stratified). Overall estimates (crude and adjusted) are displayed as purple diamonds; all subgroup estimates are displayed as teal diamonds with horizontal error bars. The vertical reference line indicates the null (*β* = 0). *p* values for interaction test the homogeneity of effect across subgroups.

The association was directionally consistent between sexes, with a marginally steeper slope in females (*β* = 2.19; 95% CI: 1.94–2.43) than males (*β* = 2.03; 95% CI: 1.79–2.27), though the sex-by-D₃ interaction was not statistically significant (*p* for interaction = 0.271). By age, the point estimate was largest in children aged 6–11 years (*β* = 4.10; 95% CI: 2.10–6.11), approximately twice the magnitude observed in younger age strata. Notably, the association in infants 0–6 months was imprecisely estimated (*β* = 1.73; 95% CI: −1.27–4.73; *n* = 29), with the wide confidence interval crossing the null, likely reflecting limited statistical power in this small subgroup. The age-by-D₃ interaction test did not reach significance (*p* = 0.358).

Seasonal variation was pronounced and statistically significant (*p* for interaction = 0.002). The association was strongest in winter (*β* = 2.70; 95% CI: 2.23–3.17) and weakest in summer (*β* = 1.79; 95% CI: 1.49–2.09), with spring (*β* = 2.03; 95% CI: 1.74–2.33) and autumn (*β* = 2.22; 95% CI: 1.84–2.61) occupying intermediate positions. This pattern is consistent with UVB-driven cutaneous D₃ synthesis during summer months raising baseline D₃ levels and thereby compressing the observable range of the D₃–retinol association.

The threshold-stratified analysis revealed a marked divergence in effect magnitude around the 28.9 ng/mL breakpoint. Below this threshold, the slope was nearly twofold steeper (*β* = 3.28; 95% CI: 2.77–3.79; *n* = 3,143) than above it (*β* = 1.66; 95% CI: 1.34–1.98; *n* = 3,631). The interaction between D₃ group (<28.9 vs. ≥28.9 ng/mL) and continuous D₃ was highly significant (*p* for interaction <0.001), formally confirming the non-linear, threshold-dependent nature of the D₃–retinol relationship. The stratum-specific slopes estimated by separate within-group regressions (Figure 2) were consistent with the piecewise regression estimates (3.158 and 1.804) but with slightly narrower confidence intervals due to subgroup-specific variance estimation.

### Tertile-stratified analysis

To further characterize the changing strength of the D₃–retinol association across the nutritional spectrum, we fitted separate linear regression models within D₃ tertiles. In the lowest tertile (*n* = 2,257), the adjusted slope was 3.11 ng/mL per 1 ng/mL D₃ increment (95% CI: 2.45–3.77; *p* < 0.001), indicating a robust positive association in the lower repletion range. In the middle tertile (*n* = 2,259), the adjusted estimate attenuated markedly to 1.02 (95% CI: −0.13–2.17; *p* = 0.083), with the confidence interval spanning the null. In the highest tertile (*n* = 2,258), the adjusted slope was 1.17 (95% CI: 0.73–1.61; *p* < 0.001), remaining statistically positive but substantially weaker than in the lowest tertile. The interaction between D₃ tertile and continuous D₃ was highly significant (*p* for interaction <0.001 in both crude and adjusted models), formally confirming that the D₃–retinol association varies non-uniformly across the nutritional spectrum. These tertile-specific estimates illustrate a gradient of association strength rather than a uniform linear relationship.

## Discussion

This analysis of an expanded pediatric cohort demonstrates a D₃-specific non-linear association with serum retinol, characterized by a steep positive relationship at lower D₃ concentrations and a progressively attenuated—but still statistically positive—association at higher concentrations. The finding refines and extends prior work describing a total 25(OH)D interaction near 28 ng/mL in a 2018–2021 Ningbo cohort. Three features distinguish the current investigation.

First, the D₃-specific dose–response profile sits marginally higher than the total-vitamin-D equivalent, and the slope below the transition region is steeper (3.16 versus 2.94 in the earlier total-D analysis). This divergence is analytically coherent: D₃ exhibits two- to three-fold greater vitamin D-binding protein (DBP) affinity and preferential hepatic 25-hydroxylation by CYP2R1 compared with D₂ (10). Consequently, a given total 25(OH)D concentration driven predominantly by D₃ reflects a more potent vitamin D signal per unit mass. The observed attenuation of the D₃–retinol association at higher concentrations therefore occurs at a slightly higher D₃ level than would be inferred from total-vitamin-D assays that average in the weaker D₂ signal. This observation supports a shift from “total-vitamin-D sufficiency” to “isoform-specific precision” in populations where D₃ monotherapy is standard (11, 12).

Second, the baseline profile of this 2019–2023 cohort reveals secular trends. Severe D₃ deficiency was rare (0.2%), roughly one-seventh the prevalence observed in the earlier cycle. This likely reflects heightened post-pandemic parental awareness of micronutrient supplementation, alongside sustained national D₃ fortification (12, 13). Nevertheless, the spring nadir persisted (42.6% of severe cases sampled in spring), confirming that seasonal metabolic memory—driven by winter UVB deprivation and D₃‘s approximately 15-day half-life—operates independently of secular improvements (14). The age gradient also persisted: deficient children were older (median 1.2 years in the deficiency group versus 0.7 years in the sufficient group), underscoring waning supplementation adherence as toddlers transition to family diets and reduce outdoor activity (15).

Third, the subgroup forest plot reveals nuanced patterns that provide clinical context. The sex-stratified analysis shows a consistent D₃–retinol association in both girls and boys, with a marginally steeper slope in females (*β* = 2.19 versus 2.03). This aligns with known sex differences in vitamin D metabolism and adipose distribution of fat-soluble vitamins, though the magnitude of the sex difference is smaller than in the earlier cohort, suggesting that secular supplementation trends may be compressing gender disparities (16). The null finding in 0–6-month infants likely reflects dominant breastfeeding, which buffers retinol fluctuations against D₃ variation. Likewise, the apparently stronger association in children aged 6–11 years (*n* = 93) should be regarded as hypothesis-generating, given the small stratum size and the non-significant age interaction. Most striking is the seasonal interaction: the winter–summer difference in effect magnitude (*β* = 2.70 versus 1.79) implies that UVB-driven D₃ synthesis not only raises baseline D₃ levels but also compresses the observable slope of the D₃–retinol association, presumably because summer cutaneous production elevates D₃ independently of dietary intake (17).

The tertile-stratified analysis offers additional granularity beyond a simple dichotomy. The robust association in the lowest tertile (*β* = 3.11), the attenuation in the middle tertile (*β* = 1.02), and the modest but significant association in the highest tertile (*β* = 1.17) collectively demonstrate that the D₃–retinol relationship is not uniform across the nutritional spectrum. Rather than a single breakpoint, the data support a continuous gradient of association strength that weakens as D₃ repletion improves. This pattern is consistent with a saturable nutritional interaction in which the marginal contribution of additional D₃ to retinol status diminishes once adequate repletion is approached.

The saturable pattern is biologically plausible. VDR signals exclusively as a heterodimer with RXR, so vitamin D-dependent transcription is constrained by retinoid availability, and the two pathways may cross-regulate most strongly at low repletion (18). In addition, D₃ and retinol share intestinal uptake and transport via chylomicrons and lipid transporters (CD36, SR-BI, NPC1L1) and hepatic RBP4-mediated secretion, which would be expected to co-vary when intake is low and to saturate as repletion improves (19).

From a public health perspective, the non-linear D₃–retinol association identifies an observational transition point (approximately 29 ng/mL) that is relevant to China’s D₃-only national program. Current guidelines often cite total 25(OH)D sufficiency (>20 ng/mL), yet our data suggest that the association with retinol status is steepest in the upper insufficient to lower sufficient range (approximately 29 ng/mL). The winter-specific strengthening of the D₃–retinol association is consistent with the hypothesis that children in low-UVB seasons might show a stronger retinol response to D₃ supplementation, as their systems operate in the steep portion of the dose–response curve; this hypothesis, however, requires confirmation in prospective or interventional studies. This isoform-specific estimate should be regarded as hypothesis-generating rather than a validated clinical target; whether maintaining D₃ above this observational transition point improves retinol status requires confirmation in prospective intervention trials. Whether this observational estimate could inform D₃ supplementation practice in children—particularly those with limited sun exposure, those examined during winter months, or those in rapid growth phases—remains to be tested in prospective or interventional studies.

Several limitations warrant caution. The cross-sectional design precludes causal inference regarding temporal sequencing. Although we adjusted for calendar year, we did not explicitly stratify by COVID-19 lockdown periods (e.g., February–April 2020 and December 2022), during which sun exposure and dietary patterns were aberrant. Unreported over-the-counter D₃ dosing frequency was not captured, limiting dose–response granularity. The retinol acute-phase response was mitigated by excluding recent infections, but subclinical inflammation without CRP elevation could still confound results. Moreover, the cohort was strongly skewed toward infancy and early childhood (median age 0.7 years; 87.1% under 3 years), whereas only 93 children (1.4%) were aged 6–11 years. The small samples in both the youngest (0–6 months, *n* = 29) and oldest (6–11 years, *n* = 93) age subgroups rendered their effect estimates imprecise, and the non-significant age-by-D₃ interaction should not be interpreted as evidence of homogeneity across age groups; the apparently stronger association in school-age children is therefore hypothesis-generating only, warranting dedicated investigation of these ages in future cohorts. Finally, the single-center coastal region (Ningbo, 29°N) with high seafood consumption limits generalizability to higher-latitude or inland populations.

## Conclusion

In this expanded 2019–2023 pediatric cohort, serum 25(OH)D₃ exhibits a non-linear, isoform-specific association with retinol, with robust evidence of a transition from steep to attenuated association near 29 ng/mL. Subgroup analyses reveal that this D₃-specific relationship operates across sexes and most age groups, with pronounced seasonal modulation—strongest in winter when UVB-driven synthesis is scarce. This isoform-specific profile provides observational evidence relevant to China’s D₃-only supplementation policy, although the ~29 ng/mL transition point should be regarded as an observational association threshold rather than a clinical supplementation target; it provides an empirical anchor for future studies aiming to integrate bone health and mucosal immunity targets. Longitudinal trials are now needed to test whether maintaining D₃ within the optimal repletion range prevents retinol insufficiency and its downstream infectious and allergic morbidities.

## Data Availability

The original contributions presented in the study are included in the article/supplementary material, further inquiries can be directed to the corresponding authors.

## References

[1] Hu Y Jiang S Lu J Yang Z Yang X Yang L. Vitamin D status for Chinese children and adolescents in CNNHS 2016–2017. Nutrients. (2022) 14:4928. doi: 10.3390/nu14224928, 36432613 PMC9693967

[2] Chen Q Liu Y Chen L Chen J Yang T Cheng Q . Vitamin a levels among pre-school children of central and Western China. Front Public Health. (2021) 9:694106. doi: 10.3389/fpubh.2021.694106, 34552903 PMC8450327

[3] Wu QH Chen Q Yang T Chen J Chen L Xiang XL . A survey on the current situation of serum vitamin a and vitamin D levels among children aged 2–<7 years of 20 cities in China. Zhonghua Er Ke Za Zhi. (2024) 62:231–8. doi: 10.3760/cma.j.cn112140-20230923-00216 38378284

[4] Giustina A Bilezikian JP Adler RA Banfi G Bikle DD Binkley NC . Consensus statement on vitamin D status assessment and supplementation: whys, Whens, and Hows. Endocr Rev. (2024) 45:625–54. doi: 10.1210/endrev/bnae009, 38676447 PMC11405507

[5] Balachandar R Pullakhandam R Kulkarni B Sachdev H. Relative efficacy of vitamin D2 and vitamin D3 in improving vitamin D status: systematic review and meta-analysis. Nutrients. (2021) 13:3328. doi: 10.3390/nu13103328, 34684328 PMC8538717

[6] Benameur T. Seasonal variations in 25-Hydroxyvitamin D levels among pediatric patients attending the healthcare Centre. Nutrients. (2024) 16:379. doi: 10.3390/nu16030379, 38337664 PMC10856968

[7] Xu C Chen X Chen Y Wen Z Cheng F. Establishment of age-specific reference intervals for serum 25-hydroxyvitamin D in a large pediatric population of Nanjing, China. Heliyon. (2024) 10:e37409. doi: 10.1016/j.heliyon.2024.e37409, 39296073 PMC11408806

[8] Zhang Y Zhou L Ren Y Zhang H Qiu W Wang H. Assessment of serum vitamin D levels in children aged 0–17 years old in a Chinese population: a comprehensive study. Sci Rep. (2024) 14:12562. doi: 10.1038/s41598-024-62305-7, 38821990 PMC11143243

[9] Liu W Wang Q Mao B Xu F Ke Y Zhang S . Two-phase linear relationship of vitamin D and vitamin A among children aged 0–14 years: a cross-sectional study. Front Nutr. (2025) 12:1539590. doi: 10.3389/fnut.2025.1539590, 40236634 PMC11996658

[10] Bouillon R Schuit F Antonio L Rastinejad F. Vitamin D binding protein: a historic overview. Front Endocrinol. (2020) 10:910. doi: 10.3389/fendo.2019.00910, 31998239 PMC6965021

[11] Van Den Heuvel EG Lips P Schoonmade L Lanham-New S Van Schoor NV. Comparison of the effect of daily vitamin D2 and vitamin D3 supplementation on serum 25-hydroxyvitamin D concentration (total 25(OH)D, 25(OH)D2, and 25(OH)D3) and importance of body mass index: a systematic review and meta-analysis. Adv Nutr. (2023) 15:100133. doi: 10.1016/j.advnut.2023.09.016 PMC10831883 37865222

[12] Yazdi ZS Streeten E Whitlatch H Montasser M Beitelshees A Taylor SI. 6652 precision diagnostic approaches for assessing clinical vitamin D status. J Endocr Soc. (2024) 8:bvae163.473. doi: 10.1210/jendso/bvae163.473

[13] Doğanay D Aydin M Aslan N Çelik İ Doğanay A Çelik Y. Parents' habits of using food supplements for their children: the impact of the COVID-19 pandemic process use of nutritional supplements in children during COVID-19. J Res Pharm. (2025) 29:335–45. doi: 10.12991/jrespharm.1644375

[14] Khanna T Shraim R Žarković M Van Weele M Van Geffen J Zgaga L. Comprehensive analysis of seasonal and geographical variation in UVB radiation relevant for vitamin D production in Europe. Nutrients. (2022) 14:5189:14. doi: 10.3390/nu14235189, 36501219 PMC9735494

[15] Qiao Y Wang X Ma Y Hu J. Variations in vitamin D status among Chinese children aged 1–6 years during the COVID-19 pandemic. Front Public Health. (2025) 13:1514355. doi: 10.3389/fpubh.2025.1514355 PMC11799289 39916704

[16] Muscogiuri G Barrea L Somma C Somma CD Laudisio D Salzano C . Sex differences of vitamin D status across BMI classes: an observational prospective cohort study. Nutrients. (2019) 11:3034. doi: 10.3390/nu11123034, 31842281 PMC6950363

[17] Scholze A Shinkov A Slavov G Slavov GS Luman M Vallianou N . 25-Hydroxyvitamin D3 metabolism modulates the effect of variable UV exposure on 25-hydroxyvitamin D3 plasma concentrations. Commun Med. (2026) 6:105. doi: 10.1038/s43856-025-01368-7, 41530331 PMC12894941

[18] Mianesaz H Nagy G Póliska S Göczi L Fadel L Bojcsuk D . Genomic regions occupied by both RARα and VDR are involved in the convergence and cooperation of retinoid and vitamin D signaling pathways. Nucleic Acids Res. (2025) 53:gkaf230. doi: 10.1093/nar/gkaf230, 40167329 PMC11959543

[19] Yuan Y Chen L. Transporters in vitamin uptake and cellular metabolism: impacts on health and disease. Life Metab. (2025) 4:loaf008. doi: 10.1093/lifemeta/loaf008, 40444179 PMC12121362

